# Teething as a Cause of Disease in Infancy

**Published:** 1914-01-15

**Authors:** Charles Herrman

**Affiliations:** New York, N. Y.


### TEETHING AS A CAUSE OF DISEASE IN INFANCY.

BY CHARLES HERRMAN, M.D., NEW YORK, N. Y.

Notwithstanding the remarkable progress that has been made in the science of medicine during the last fifty years, certain traditional beliefs remain as deeply rooted as ever. Not the least noteworthy is the important role which teething is supposed to play in the etiology of the diseases of infancy. Every day mothers bring their babies to the clinic, attributing their illness to teething. Every day, in taking the history of the child, the mother volunteers the information that some previous illness was ascribed by the attending physician to teething. But it is not only in the dispensary practice that we meet with these beliefs. In private practice even very intelligent mothers are only too ready to attribute many disturbances to teething; and for this we physicians are to some extent responsible. We are so apt to follow the line of least resistance. It is so much easier to agree than to disagree and explain. Frequently we are at a loss to make a diagnosis; the mother suggests teething and we gratefully accept the suggestion.

But as Holt says, "the physician who starts out with the idea that in infants dentition may produce all symptoms, gets no further than this in his etiological investigations.

The present views with regard to teething are three: First, there are those who believe that very many disturbances may be due to teething; second, those who believe that none are due to teething; and, third, those who take a middle course, believing that a few disturbances may be caused by teething. To the first group belong a large party of the laity and some physicians. In a recent paper, Neumann has shown in graphic form that in Berlin, from 1877 to 1910, the number of certificates giving teething as a cause of death in infants has diminished from fourteen to three per 1,000. Three in a thousand is, I think, still pretty high. The number of certificates giving teething as a cause of death is an index of the intelligence of the physicians, their knowledge of pediatrics being in inverse proportion to the number of such certificates submitted.

The chief danger in believing that many disturbances are due to teething lies in the fact that valuable time may be lost. For example, in cases of acute gastroenteritis, the disease, falsely attributed to teething, is allowed to progress and when the child is dangerously ill, possibly beyond all medical help, it is brought for treatment.

The second view is that "teething produces nothing but teeth," that the eruption of the teeth is a physiological process similar to the growth of hair and nails. This view, expressed by Politzer and Fleischmann forty years ago, and more recently by Kassowitz and others, is not incompatible in a somewhat modified form with the third view. So modified it would read, teething produces nothing but teeth in normal healthy infants. In such individuals the hair and nails are of normal appearance; but just as in certain constitutional diseases, such as syphilis and cretinism, these parts may show changes, so in infants with certain constitutional peculiarities the eruption of teeth may be sometimes associated with certain disturbances. The fact that an infant has such disturbances is, I believe, proof that it is not normal. I shall elaborate this point later.

Let us consider briefly the ways in which disturbances may be incorrectly attributed to teething: First, the presence of symptoms at the time of teething may be merely a coincidence. We have all seen numbers of children go through teething without any disturbances. If a baby contracted whooping cough during dentition no one would think of holding the teeth responsible, because we know that this disease is caused by a specific infectious material and is acquired by contact with another individual having the disease. The eruption of the twenty temporary teeth usually takes place from the seventh month to the beginning of the third year, that is, on the average about one tooth a month. It is therefore not surprising that disturbances quite independent of teething should occasionally occur when a tooth is appearing. Many babies are weaned at the seventh month; what is more natural than that the child should have digestive disturbances when put upon artificial feeding? Second, the true cause of the disturbance may not be recognized on account of an incomplete examination. For example a baby is restless and has a rise of temperature there is no reason to suspect ear trouble, and the ears are not examined. A diagnosis of "teething" is made and a couple of days later the drum perforates and there is a discharge. Or with similar symptoms, the urine is not examined on account of the difficulty of obtaining a specimen. Teething is diagnosticated. The symptoms persist, the urine is examined, and a pyelocystitis is found. Third, it may not be possible to determine the cause, even with the most careful and painstaking examination. It occasionally happens that even at the autopsy table the cause of death cannot be ascertained, but we are not on that account justified in attributing it to teething.

Teething produces nothing but teeth in normal, healthy infants, but many infants have constitutional peculiarities which are not strikingly apparent. Children with mild manifestations of rickets may have disturbances of dentition, but there are two constitutional peculiarities to which especially I should like to call your attention, namely, the so-called exudative and the spasmophilic diatheses.

There is a form of papular urticaria frequently met with in infants. This has often been attributed to teething, as the German name, *Zahnpocken*, indicates. This eruption is but one of the manifestations of the exudative diathesis. Certain forms of infantile ecxema are due to the same cause and are falsely ascribed to teething. These children with the exudative diathesis are also subject to recurring attacks of catarrh of the mucous membranes, rhinitis, laryngitis, bronchitis. I have also seen a number of such infants with recurring attacks of diarrhea with loose, mucous stools, quite independent of indiscretions of diet. Should such an attack occur at the time of the eruption of a tooth, what would be more natural than to suppose that one was the cause of the other? A careful analysis of the previous history, however, together with a further observation of the patient, will reveal the true underlying etiological factor, namely, this peculiar constitutional anomaly which is probably due to some disturbance of metabolism, the exact nature of which is as yet unknown.

Similarly the convulsions occurring in infancy are frequently ascribed to teething, as witness the German term *Zahnkraempfe*. We now know, however, that such children have the so-called spasmophilic tendency, and even in those who do not give all the clinical symptoms associated with this condition, the electrical reactions present changes from the normal. Moro, as evidence of a possible causal connection, says: "The occurrence of genuine eclamptic seizures has been observed in some infants during the period of teething and never afterward." But it must be remembered that during the period of dentition many other changes are taking place; it is a time of rapid development of nearly all the structures of the organism. In this connection it is noteworthy that the manifestations of the exudative and spasmophilic diathesis have a tendency to diminish or disappear as the child grows older. "Outgrowing a disease" is not merely a phrase, in these conditions it actually takes place.

Many of the symptoms attributed to teething are met with in the early months, before the eruption of teeth has begun. Many mothers consider an increased flow of saliva and putting the fingers in the mouth indications of the onset of teething; but I have seen countless babies in which both these symptoms were present quite independently of dentition. The eruption of the permanent teeth is occasionally attended with some disturbance, but how often would one attribute a skin eruption, a diarrhea, or a convulsion in an older child to teething? At a time when teething was considered the cause of a number of infantile disorders, the gums were frequently lanced. Why has this operation been so largely given up? Obviously because it did not meet the indications. It is still done occasionally, but I believe often, just as the cutting of the frenum of the tongue, to ease the mind of the mother.

Let us admit that a few slight disturbances may be caused by teething; that requires no emphasizing. What we should emphasize is that in the presence of a disease in an infant we should not diagnosticate teething as a cause except as a last resort, after every other possible explanation has been excluded. The teeth should be treated as we treat prisoners at the bar. We should consider them innocent until proved guilty.—*New York Medical Journal.*

